# Assessing Gait in Parkinson’s Disease Using Wearable Motion Sensors: A Systematic Review

**DOI:** 10.3390/diseases7010018

**Published:** 2019-02-05

**Authors:** Lorenzo Brognara, Pierpaolo Palumbo, Bernd Grimm, Luca Palmerini

**Affiliations:** 1 Department of Biomedical and Neuromotor Science, University of Bologna, Via Ugo Foscolo 7, 40123 Bologna, Italy; 2 Department of Electrical, Electronic, and Information Engineering “Guglielmo Marconi”, University of Bologna, Viale Risorgimento 2, 40136 Bologna, Italy; pierpaolo.palumbo@unibo.it (P.P.); luca.palmerini@unibo.it (L.P.); 3 Sylvia Lawry Centre, The Human Motion Institute, 81677 Munich, Germany; bp.grimm@gmail.com

**Keywords:** Parkinson’s disease, gait, wearable sensor, accelerometry, inertial sensor, wearable device

## Abstract

Abstract: Parkinson’s disease (PD) is a progressive neurodegenerative disorder. Gait impairments are common among people with PD. Wearable sensor systems can be used for gait analysis by providing spatio-temporal parameters useful to investigate the progression of gait problems in Parkinson disease. However, various methods and tools with very high variability have been developed. The aim of this study is to review published articles of the last 10 years (from 2008 to 2018) concerning the application of wearable sensors to assess spatio-temporal parameters of gait in patients with PD. We focus on inertial sensors used for gait analysis in the clinical environment (i.e., we do not cover the use of inertial sensors to monitor walking or general activities at home, in unsupervised environments). **Materials and Methods:** Relevant articles were searched in the Medline database using Pubmed. **Results and Discussion:** Two hundred ninety-four articles were initially identified while searching the scientific literature regarding this topic. Thirty-six articles were selected and included in this review. **Conclusion:** Wearable motion sensors are useful, non-invasive, low-cost, and objective tools that are being extensively used to perform gait analysis on PD patients. Being able to diagnose and monitor the progression of PD patients makes wearable sensors very useful to evaluate clinical efficacy before and after therapeutic interventions. However, there is no uniformity in the use of wearable sensors in terms of: number of sensors, positioning, chosen parameters, and other characteristics. Future research should focus on standardizing the measurement setup and selecting which spatio-temporal parameters are the most informative to analyze gait in PD. These parameters should be provided as standard assessments in all studies to increase replicability and comparability of results.

## 1. Introduction

Parkinson’s disease (PD) is a chronic neurological disease characterized by deterioration of the dopaminergic neurons in the brain. Patients suffering from this disease manifest disorders that affect motor behavior and compromise the quality of life.

Motor symptoms and gait impairments are among the issues that most affect the quality of life of patients with Parkinson’s disease. PD patients can exhibit slow gait with less foot clearance and smaller step lengths with respect to healthy individuals [1,2]). PD patients consider the progressive loss of motor autonomy the first and most worrying symptomatology [3,4]. This is one of the reasons why having effective and reliable tools for gait analysis is paramount. Wearable sensor systems can aid in gait analysis by providing spatio-temporal parameters useful to investigate the progression of gait problems in PD, without the need of a specialized laboratory for movement analysis. However, various methods and tools, with very high variability, have been developed.

The aim of this study was to review published articles of the last 10 years (from 2008 to 2018) concerning the application of wearable sensors to assess spatio-temporal parameters of gait in patients with Parkinson’s disease. In this review we focus on using inertial sensors for gait analysis in the clinic environment. Therefore, we do not cover other possibly interesting fields of application of wearable sensors such as monitoring activities at home of daily living (ADL), measuring symptoms, or on–off periods [5].

### 1.1. Inertial Measuring Units

**Figure 1:**
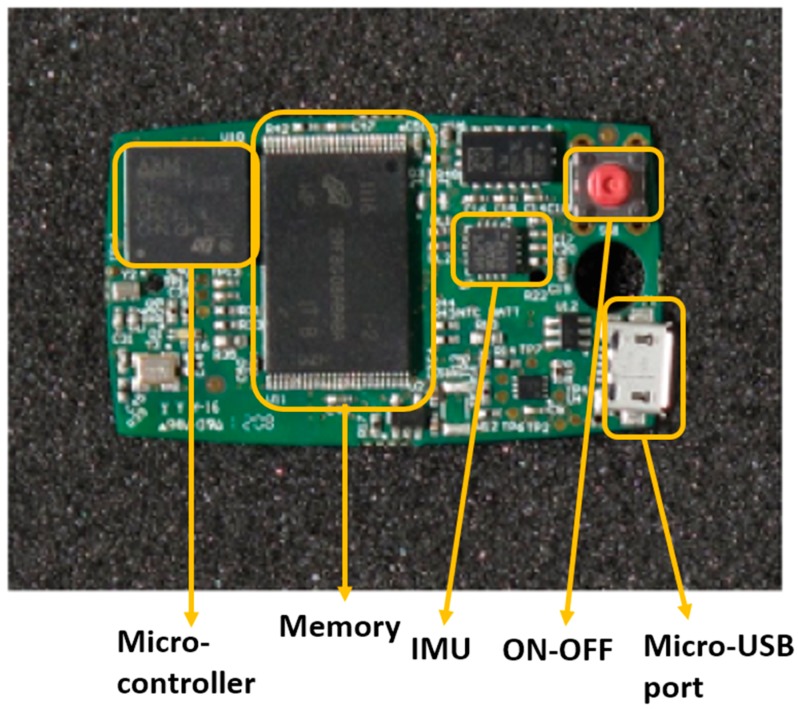
Electronic board of a wearable sensor with an inertial measurement unit (IMU).

Accelerometers can measure the acceleration of a body in by determining the inertia of a mass when it is subjected to external force and acceleration. The device is composed of a transducer, known as a micro-electro-mechanical system (MEMS) that detects movement and transforms the mechanical signal into an electric one. This tool can be used to determine accelerations and, via signal integration, speed and displacements. Besides dynamic accelerations, accelerometers also measure the static gravitational acceleration “g”. This way the device orientation related to gravity can be derived using simple trigonometry (“inclinometer” function). Based on their electro-mechanical principle, accelerometers can be classified as piezoresistive, piezoelectric, capacitive, and extensimetric. Commonly, three uniaxial accelerometers are integrated into a single 3D accelerometer to measure three orthogonal axes simultaneously. Very often, in inertial measurement units (IMUs) the accelerometer is combined with a gyroscope. The gyroscope is another MEMS device that measures angular velocities around a predefined axis, in practice most often implemented as a 3D system measuring angular velocities for three orthogonal axes (yaw, pitch, and roll). Using different on their electro-mechanical principles, gyroscopes can be electrostatic, magnetic, or cryogenic. In several types of IMUs, a 3D-magnetometer is also added in sensor fusion and then can include on-board fusion algorithms (e.g., Kalman filters). IMUs, thanks to the reduced size and costs of their components, are easy to wear and low-cost tools for movement analysis. The properties of these measurement systems have revolutionized the functional analysis allowing an objective movement analysis of patients with neurological diseases, both in clinical practice and at home [6,7]. IMUs, and the sensors they are composed of (accelerometer, gyroscope, magnetometer), can allow to estimate with great accuracy the kinematic parameters as well as the position, the acceleration, and the speed produced by the movement. In Figure 1 there is an example of an electronic board of a wearable sensor node containing an IMU. The figure is adapted from Casamassima et al. [8]. The node includes a microcontroller, an IMU (combination of accelerometer, gyroscope, and magnetometer), flash memory for local data storage, and a micro-USB port to recharge the battery.

### 1.2. Clinical Application

The walking of patients with PD is characterized by alterations (with respect to healthy subjects) of the most important spatio-temporal gait parameters such as gait velocity, cadence, stride time, and length, parameters also known to degrade with disease progression. The assessment of walking is part of the most common PD rating scales such as the Unified Parkinson’s Disease Rating Scale (UPDRS), Hoehn and Yahr (H&Y) staging, and the Schwab and England rating of activities of daily living. Recent technological developments provided clinicians with new tools to evaluate treatments in a more objective way. In the last few years there has been a growing interest for developing technologies and methods for enabling human motion analysis. Specialists are focusing more than ever on products that perform a clinical and biomechanical evaluation in a way that is as much as possible cheap, fast, and effective. Gait analysis using wearable sensors is expected to play an increasingly important role in neurology and other clinical fields. By using inertial sensors, it is possible to detect and characterize specific movements and register variations in the clinic (supervised by a clinician) and monitor activities of daily living of people with Parkinson’s disease in their own home (unsupervised). In this review we focus on using inertial sensors for gait analysis in the clinic environment (i.e., we do not cover the use of inertial sensors to monitor walking or general activities at home, in unsupervised environments where the registration of freezing or tremor event frequency, duration, and intensity may also be a useful wearable sensor application). We want to provide an overview of the recent (last 10 years) state of the art in this field, characterizing current usage and possible limitations. 

## 2. Materials and Methods

### Review Process: Search Strategy and Selection Criteria

An electronic search was performed in Pubmed on 26 October 2018 using the following search string: (parkinson*[Title/Abstract]) AND (accelerat*[Title/Abstract] OR acceleromet*[Title/Abstract] OR inertia*[Title/Abstract] OR gyroscop*[Title/Abstract] OR “wearable sensor*”[Title/Abstract] OR “body-fixed sensor*”[Title/Abstract]) AND (gait*[Title/Abstract] OR walk*[Title/Abstract]) AND (“2008/01/01”[PDAT]: “3000”[PDAT]).

**Figure 2:**
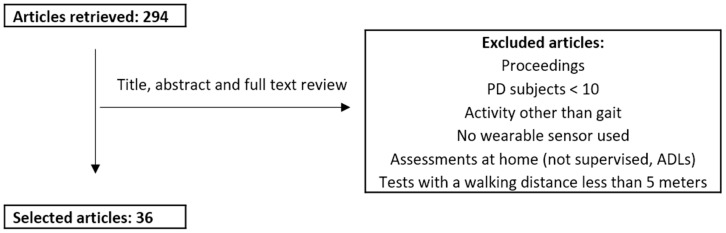
Review process. PD: Parkinson’s disease.

We excluded conference proceedings, articles reporting results from less than 10 PD patients, that did not assess gait, or that assessed gait over a walking distance shorter than five meters. As a note, “3000” as year of end is the default PubMed option when performing a search until the current day. Figure 2 presents a flowchart of the review process.

## 3. Results

### 3.1. Format and Disambiguation

Our search selected 36 articles. They are summarized in Table 1. We reported the most commonly derived parameters for spatio-temporal gait analysis. Regarding sensor locations, we grouped together IMUs on the lower part of the leg but listed sensors on foot or feet separately. The Hoehn and Yahr scale (H&Y) is provided as a clinical rating scale evaluating progression in Parkinson’s disease stages. A better consistency and standardization in placing and in reporting should be done in the future. For example, “shin” and “shank” refer to the same part of the leg: the tibia. Also, placement on the shins, ankles and on the feet can be ambiguous and mean different relative positions (lateral, front, different height or relative positions). Furthermore, the asymmetry column is checked if in the article there is at least one parameter related to the right–left difference of one of the parameters in the table (thus, asymmetry of right-left arm, which is present in some papers, is not considered in Table 1).

### 3.2. Sensor Number and Placement

Different numbers of wearable sensors were used and placed on different parts of body, as shown in Table 1. 

**Figure 3:**
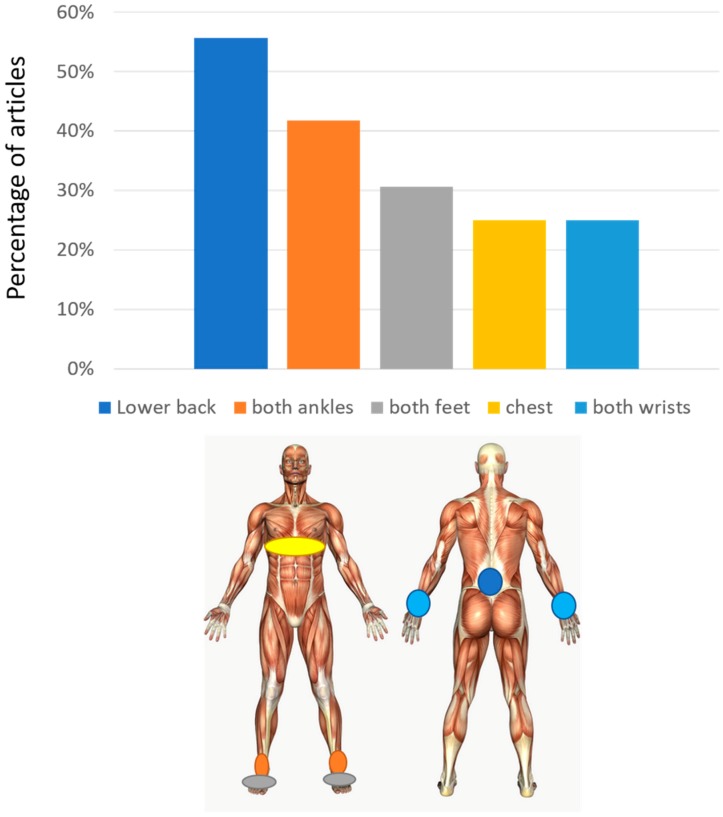
Sensor placement. At the top, the percentage of studies involving a sensor on different positions is reported. At the bottom, the corresponding position of sensors with respect to the body is reported.

The average number of sensors used in these studies is 3.2 ± 2.4 (± one standard deviation) with a minimum of 1 and a maximum of 8. The most used set-up is with a single sensor (13/36). When a single sensor is used, this is most frequently worn on the lower back (8/36). The lower back position (alone or with other sensors) is used in total in 55.6% of the studies. Other common positions are the position on both ankles (or tibias) and on both feet, found respectively in 41.7% and 30.6% of the articles. In many studies the position on the ankles/shins/shanks or feet are mutually exclusive, showing that they are mostly considered as alternative for gait analysis. The chest position is also present (25%). The chest and lower back are used both together and as alternatives. They both reflect trunk movement. Sensors are also placed on wrists in some papers, mostly to detect arm swing and its right-left asymmetry (Figure 3). 

In this review we did not find one particular multi-sensor set-up that is used by most of the studies but a wide variety of combinations. A consensus in this regard in the clinical research community is still to be gained, in terms of sensor numbers and positions, probably in a trade-off between the number and accuracy of gait parameters and clinical usability. Thus, related themes that would deserve further investigation are accuracy, redundancy, and comfort of different positions. In general, research work on what is the minimum set-up to guarantee a certain level of accuracy on a specific set of parameters would be very useful for clinical research.

### 3.3. Spatio-Temporal Parameters

**Figure 4:**
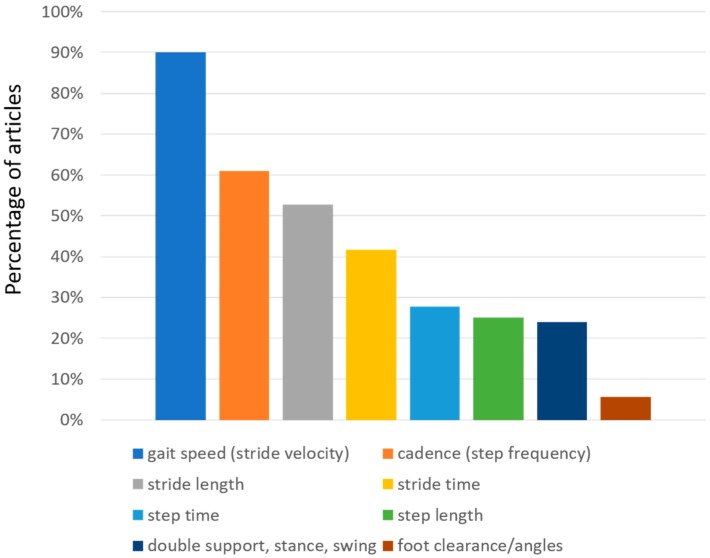
Distribution of gait spatio-temporal parameters evaluated in the selected articles. The percentage of the articles that report each parameter is presented.

**Figure 5:**
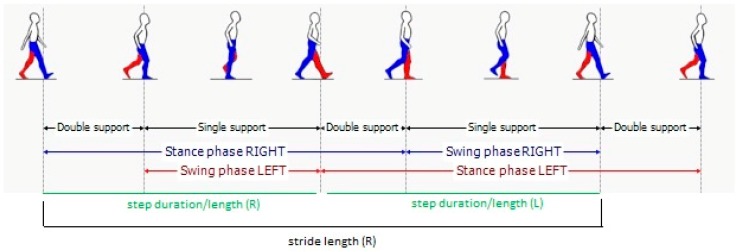
Gait phases.

Regarding the gait spatiotemporal parameters, significant differences were found between the selected articles, as indicated in Table 1. This is clearly related to the different set-up (number and positions of sensors that were used). As a clarifying example, we found that foot clearance was only calculated in two studies which used sensors on the foot. As expected, and as shown in Figure 4, gait speed is the most common parameter reported in these studies (although not in all studies), appearing in almost 90% of them. Other studies reported stride velocity, which should reflect the same underlying parameter. We grouped together the two in the table. Cadence, which is also reported as step frequency, is present in 61% of the studies. Stride length, which can be particularly useful to evaluate PD patients (who often may exhibit short steps), is present in 52.8% of the studies. Stride time, which is also referred as gait cycle duration, has been calculated in 41.7% of the studies. Step characteristics are also often evaluated, sometimes in addition to stride characteristics and sometimes in alternative. Step time and step length are reported by 27.8% and 25% of the studies, respectively. Variability of both stride and step characteristics is reported less than the normal value. Among variabilities, stride time variability is the most reported one (22.2% of the articles), followed by the (related) variability of step time (11.1%), and the variabilities of step length (8.3%) and stride length (5.6%). Right-left asymmetry is also reported for at least one of the parameters in the table (16.7%). Very specific parameters related to the spatial position of the foot (foot clearance, heel strike and toe off angles) are reported only in two studies (5.6%). Instead, time parameters related to the characteristics of the gait cycle (Figure 5) like double support, stance, and swing time are reported between 22% and 25% of the times. These parameters are reported as percentages or as durations (seconds). In the majority of studies, the spatiotemporal gait parameters derived were known or derivates of metrics already established in gait laboratory assessments using e.g., video-capture. Thus, the parameters’ value for outcome assessment or diagnostics was largely assumed as given. Novel parameters enabled by the new sensor modalities such as for example gait complexity measures [43] or body segment coordination variability [44] have not yet been explored much and would require a more systematic validation approach for its construct, discriminative power, and minimal clinically important difference (MCID), or as a clinical study endpoint.

### 3.4. Characteristics of Patients

Studies included an average of 51.3 ± 48.8 patients, with a minimum of 10 and maximum of 190. We excluded papers based on less than ten patients. Most studies (72.2%) reported Hoehn and Yahr staging of disease, so it is recommended that new studies report this value. Some of the studies additionally (or alternatively) report the motor score of the Unified Parkinson’s Disease Rating Scale (UPDRS), which can indeed be useful to further characterize the cohort of patients. Unfortunately, some studies did not report these values at all, making it difficult to draw conclusions on effectiveness or to compare with other studies.

## 4. Discussion

Besides the reported and structured analysis based on Table 1, we also observed further promising approaches for assessing PD patients using wearable IMUs, that could be relevant for further investigation. First, besides the reported parameters, which are the most common in studies performing gait analysis, other potentially meaningful parameters were also collected in some of the reported studies. As an example, besides the already reported arm swing asymmetry, these were: stride regularity, harmonic ratio, and turning characteristics (for the tests that included turning). With motor asymmetry being a characteristic of PD-related abnormalities in locomotion [45], bilateral measurements and asymmetry parameters seem promising disease-specific sensor metrics. Thus, obviously our review is not exhaustive and further work may be done for other sets of parameters.

Regarding the type of gait tests, we excluded the ones with a walking distance of less than 5 meters in order to obtain only tests where continuous walking was less affected by the start and stop phases of gait. We observed a variability in the distance walked and in the type of gait test, which may be an important aspect to investigate further which could also benefit from standardization.

Finally, some papers report reference values for their parameters, which could aid the exchange and comparison of results, while others do not. Some also present values from healthy controls, which can help in evaluating the usefulness of parameters in characterizing PD impairments. Furthermore, some papers perform comparison with gold standards (or use methods that were previously compared with gold standard technologies), while others do not. This may also impact on reliability and comparison across results. It is clear that every IMU system used to quantify gait in PD should have been previously validated in PD patients with respect to a gold standard such as an optical motion capturing system.

## 5. Conclusions

This review aimed at showing the state of the art in performing gait analysis with wearable sensors and the related variability across studies regarding characteristics such as the number and positions of the sensors, the reported spatio-temporal parameters, and so on. Consensus on the assessment protocols and selected parameters is still missing with wearable technology, which is a new technology still maturing. Still, it offers the possibility to be used in many applications and it provides the assessment of known and novel digital gait parameters. This review can be seen as a first step toward defining a consensus set-up (or a number of set-ups that can be suggested for different parameters), and a minimum subset of parameters that should be present in all studies. Furthermore, several points to increase standardization, replicability, and comparison across studies were raised.

Despite the fact that we considered only relatively recent articles, we observed a very high variability in the number and positions of these wearable sensor. A single common set-up was not prominent. Set-ups with between one and eight sensors, in different positions, were used. However, we found that the most used set-up was with a single sensor on the lower back, which obviously poses a trade-off in the number of spatio-temporal parameters that can be effectively recorded (e.g., it would not be possible to report foot clearance). Further research is needed to recommend the best set-up (or the best set-ups if two or more set-ups are equivalent) for assessing a set of spatio-temporal parameters. Regarding this, we also found a very high variability concerning the different spatio-temporal parameters reported. Also, further research should be performed linking the position of sensors and the parameters that can be obtained from those positions. Parameters should be evaluated with respect to their usefulness in clinical practice. Thus, the choice of number of sensors and sensor location(s) should be driven by the clinical relevance and required accuracy of the specific gait parameters and the usability of the set-up. Gait speed, cadence, and stride length were the most reported parameters; further work should be done to choose the most effective parameters for different aims (e.g., to evaluate fall risk, to evaluate efficacy of a treatment, to evaluate difference between health and PD etc.). In a patient-centric approach the development of wearable sensor assessment protocols and derived outcome parameters should consider the usability aspects of the test as well as the impact of the derived outcome metrics for the patients in order to establish the method in clinical routine. In such an approach, the wearable sensor technology’s capacity to quantify upper extremity locomotion (which was not the focus of the present paper) could also be evaluated, independent by itself or simultaneous and in relation to gait.

Cohorts of patients present in the studies were up to 190 patients, with an average of more than 50 patients. Recently there has been a number of studies with a relatively high number of PD patients using wearable sensors, showing increasing usage and interest in clinics and research. The presence of healthy matched subjects (not present in all studies) would be advisable. The characterization of patients with at least the H&Y stage is advisable, preferably adding further information such as the UPDRS motor score.

To summarize, approaches to gait analysis in PD patients are various regarding the type of sensor, sensor location, measurement duration/length, gait parameters, and clinical measures of patients. No consensus is present yet. Currently in the literature there is not enough evidence to support an informed clinical decision about the best system characteristics (number and positions of sensors) to choose to obtain effective clinical outcomes.

## Figures and Tables

**Table 1:** Characteristics of wearable sensors and parameters. H&Y: Hoehn and Yahr.

Ref	# PD subjects	H&Y stage	IMUs on both Ankles or on both Tibias	IMUs on both Feet	IMU on Lower Back	Other Locations (#IMUs)	# IMUs	Gait speed (stride velocity)	Cadence (or Step Frequency)	Stride Length	Stride Length Variability	Stride Time (Gait Cycle Time)	Stride Time Variability (Gait Cycle Time Variability)	Step Length	Step Length Variability	Step Time	Step Time Variability	Asymmetry Right-Left	Double Support (Time or %)	Stance (Time or %)	Swing (Time or %)	Foot Clearance	Heel-Strike and Toe-Off Angles
[9]	51	2–4				knees (2)	2	x		x		x		x		x							
[10]	51		x		x		3	x		x		x											
[11]	27	1–3	x	x	x	thighs (2), chest (1)	8	x				x	x	x	x	x	x	x					
[12]	50	1–3		x			2	x	x	x									x	x	x		
[13]	22	2.5–3.5		x			2	x	x	x		x				x			x	x	x		
[14]	125		x				2	x	x	x		x							x	x	x		
[15]	50	2–3		x			1	x	x	x												x	x
[6]	190	2.12 ± 0.06		x			2	x	x	x		x								x	x	x	x
[16]	140		x			hip (1)	3	x					x										
[17]	43		x		x		3	x					x			x		x					
[18]	12	1–3	x		x	wrists (2), chest (1)	6	x	x	x		x											
[19]	56		x	x	x	wrist (2), chest (1)	8	x		x													
[20]	28	2.35 ± 0.5	x	x	x	wrist (2)	7		x	x								x	x				
[21]	14	1–3				ankle (1)	1	x	x				x	x									
[23]	104	2.5 ± 0.6	x		x	wrists (2), chest (1)	6	x	x	x		x							x	x	x		
[24]	14	1.77 ± 0.44	x		x	wrists (2)	5	x						x	x								
[25]	124	1–4: 1 (13), 2 (31), 3 (68), 4 (12).				waist (1)	1	x	x					x		x	x						
[26]	100	ON 2.33 ± 0.53, OFF 2.51 ± 0.57	x		x	wrists (2), chest (1).	6	x	x														
[27]	39	2–3		x	x		3	x	x				x					x	x				
[28]	30	2–3: 2 (15), 3 (15)				hip (1)	1	x															
[29]	16	1–3: 1 (2), 2(8), 3 (6)		x			2	x		x		x											
[30]	10				x		1	x				x				x				x	x		
[31]	104	2–4: 2 (52), 3–4 (52)	x		x	wrists (2), chest (1)	6	x	x	x		x											
[32]	30	1–3: 1 (8), 2(20), 3 (2)			x		1							x	x	x	x	x		x	x		
[33]	10					head (1)	1	x	x					x									
[34]	12	2–4	x	x		thighs (2)	6	x	x	x		x						x	x	x	x		
[35]	110	1–4			x		1	x				x	x			x							
[36]	14				x		1	x	x	x													
[37]	20	1.5–2.5: 1.5 (1), 2 (1), 2.5 (18)			x		1									x	x						
[38]	24				x		1	x	x	x													
[39]	13				x		1	x	x	x													
[40]	12	1–2.5	x			wrists (2), thighs (2), chest (1)	7		x										x	x			
[41]	153	2–4: 2 (71), 3 (64), 4 (18)				legs (2), chest (3)	5	x	x					x									
[2]	12	1–2.5	x			wrists (2), chest (1)	5	x	x	x	x	x	x						x				
[42]	11	1–3			x		1	x	x	x	x	x	x										
Sum			15	11	20			32	22	19	2	15	8	9	3	10	4	6	9	9	8	2	2
